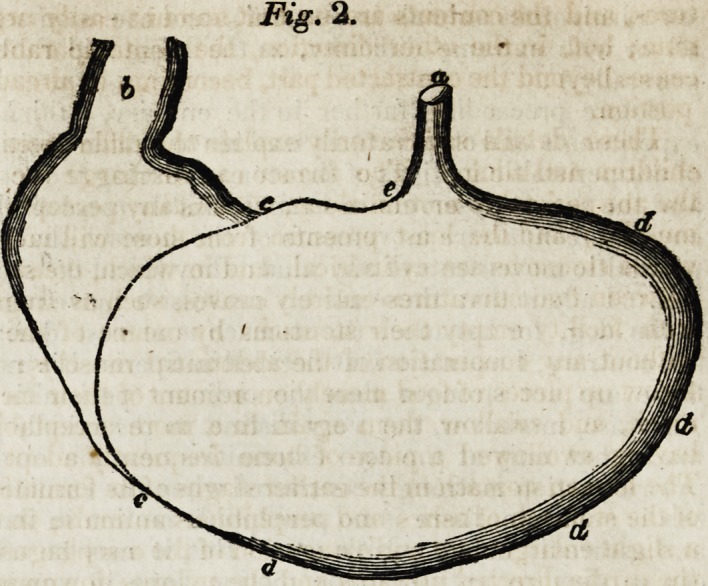# Physiology

**Published:** 1836-10

**Authors:** 


### PHYSIOLOGY.

### *On the Physiology of Vomiting; and on the Causes of its Difference in Adults and Children.* By Professor C. H. Schultz, M.D.

The great frequency of vomiting in infants at the breast, and the spontaneousness and facility with which this process takes place, are well known. It seems to occur without any previous nausea, as the infants, generally speaking, exhibit no signs of uneasiness. The case, as is well known, is very different with adults, in whom nausea and retching will, in certain cases, exist in a great degree for days, or even weeks, without any evacuation of the contents of the stomach. The facility of vomiting in general remains with children for some years after weaning, although this is effected with somewhat greater difficulty than during the period of nursing. The causes of this difference in the readiness to vomit at different ages has not, as far as I know, been yet closely investigated.

To enable us to prosecute this inquiry with advantage, it is necessary that we should have a perfect understanding of the causes of vomiting in general; and to this point I shall address myself in the first place.

The opinion first advanced by Boyle, that, in the act of vomiting, the stomach is passive,—the evacuation of its contents being effected by the contemporaneous contraction of the abdominal muscles and diaphragm,—has been adopted and powerfully advocated by physiologists of the greatest name, more especially of late years.

Chirac confirmed the fact stated by Boyle, that no convulsive motions are felt in the stomach during vomiting in the case of dogs, when the hand is placed in contact with the organ through a wound made in the abdomen. Van Swieten, Senac, and others, adopted the opinion of Boyle on other grounds; and, in later times, Magendie has proved beyond question, that, in the case of dogs, not only are no convulsive motions of the stomach *felt* during vomiting, but none are *seen* when the stomach is laid bare; and, moreover, that when the abdominal muscles are removed, and the contractile power of the diaphragm destroyed, the act of vomiting in dogs, if not entirely prevented, is, at least, rendered extremely difficult. It accords with this view of the process that, in man, vomiting becomes easier in proportion as the stomach is distended, and is thus more exposed to compression between the above-named muscles.

The objection to this explanation, derived from the fact that vomiting takes place in birds and amphibia which have no diaphragm, as also in certain cases in the human subject in which an abnormal position of the stomach had removed it from the pressure of this muscle, is not valid, since in such cases the thoracic viscera, during inspiration, present sufficient resistance to allow the stomach to be compressed between them and the abdominal muscles. It is indeed obvious, that the same muscular action takes place in the act of vomiting as in labour, cough, and the evacuation of the bowels and bladder, &c.; and that the discharge of the contents of the stomach by repeated fits or impulses, corresponds exactly with the spasm-like contractions of the abdominal muscles and diaphragm.

It has not, however, escaped the opposers of Magendie's theory, that if vomiting were effected exclusively by the abdominal muscles and diaphragm, it ought to be a purely voluntary act; whereas, it is known that only very few animals, such as frogs and birds of prey, can evacuate the contents of the stomach at pleasure. It results from this fact alone, that the before-mentioned muscles are not exclusively those which are active during vomiting; and we are hence led back to the old doctrine of the anti-peristaltic motion of the digestive organs. Maignault and Beclard have attempted to prove that, although the stomach is not spasmodically contracted, still that the œsophagus is thus affected, by fits, during vomiting in the dog; and every one who has experienced vomiting in his own person must have felt that these reverse spasmodic efforts of the muscles of deglutition commence in the pharynx. These gentlemen were further of opinion that, in the act of vomiting, no anti-peristaltic movements take place in the stomach, but that this organ presents a state of equable tonic contraction, and that it is only by means of the fitful contractions and expansions of the œsophagus, aided by the action of the abdominal muscles, that the stomach is emptied of its contents.

While acknowledging our obligations to the French investigators, we must admit that there are many phenomena attending the act of vomiting which prove their theory to be at least insufficient. If the œsophagus and abdominal muscles are the only parts active during vomiting, how is the phenomena of fæcal vomiting to be explained? I consider this morbid state sufficient proof in itself that an anti-peristaltic action both of the intestinal canal and stomach does exist, while, on the other hand, no one can deny that there may and do exist contractions of the abdominal muscles, diaphragm, and œsophagus, without any vomiting. This is evident in the case of the horse, rabbit, hare, guinea-pig, and several other herbivorous animals, which cannot be made to vomit even by the strongest emetics, although the strongest retching and contractions of the abdominal muscles take place, and although they possess the same organs as the dog, which vomits on the slightest occasion. It is the more important to investigate the cause of this difference in animals, as it will lead to the explanation of the much greater facility of vomiting in children than in adults.

The cause of these differences lies in the particular shape of the stomach in different animals, a circumstance, as far as I know, hitherto unnoticed by comparative anatomists; and the same cause operates in producing the difference in the facility of vomiting in the infant and the adult; since there exists the same analogous difference of form between the stomach of the child and the adult man, as between the stomach of animals which vomit with facility, such as the dog and cat (and we may say carnivorous animals in general), and the stomach of those which vomit not at all or with extreme difficulty, as the horse and rabbit, (and herbivorous animals generally.)

Before proceeding further in the enquiry, I think it necessary to state that my experiments and observations lead me to decide positively in favour of the existence of antiperistaltic motions of the stomach during the act of vomiting. Boyle, Chirac, and the recent observers in France, hastily concluded that, because they could discover no *convulsive* movements of the stomach that therefore there were no antiperistaltic movements of any kind: they found the stomach contracted and motionless. I admit that there are no convulsive movements, but I cannot concede that in the dog, for instance, the stomach is at rest during the act of vomiting. On the contrary, I maintain that decided antiperistaltic movements are perceptible, but these are not stronger than the ordinary peristaltic motions of the same organ. They are, moreover, not very distinct in the middle portion and fundus of the stomach, but only at the two extremities near the cardia and pylorus. The whole pyloric portion is strongly contracted when the cardiac portion expands; and, while this is going on, there is no perceptible motion in the fundus and larger curvature, and assuredly no convulsive one. But, it may be asked, what considerable effect can so slow an antiperistaltic motion have in vomiting? The answer is briefly this,—that, by this anti-peristaltic motion, (no doubt assisted by the abdominal muscles,) the *direction* is given to the food which is to be ejected by the act of vomiting, or which is to be forced from the intestines into the stomach in the case of fæcal vomiting. If the abdominal muscles alone acted on the perfectly passive stomach, the food might, by this pressure, be driven into the intestine as well as into the œsophagus; if, then, the contents of the stomach are to be ejected in a particular direction, it is requisite that the cardiac and pyloric portions should possess a distinct active motion.

I now return to the various forms of the stomach occasioning the differences in vomiting: and here I may take for granted as understood what I have detailed in the work '*De Alimentorum Concoctione*,' concerning the forms of the stomachs of carnivorous and herbivorous animals. It is demonstrable that a child's stomach is as different from that of an adult as a pole-cat's is from that of a rat; and, if the difference between the form of a child's stomach and that of an adult has not been sooner recognised, it is only because their very different functions and importance in the preservation of life had not previously been suspected; for this difference will not fail to strike every one as soon as his attention is directed to it. But, to make these differences still more conspicuous, I will introduce an outline of the form of a child's stomach, and that of an adult.

*Fig.* 1.

The stomach of a child (*Fig.* 1.) is more of a conical form, drawn out lengthwise, and gradually narrowing towards the two extremities, inferiorly towards the pylorus (*b*), superiorly towards the cardia (*a*). The œsophagus is inserted into the fundus at the left extremity, and at a distance from the pylorus; the small curvature is stretched out lengthwise (*c*), the large curvature (*dd*) is less developed, and runs almost parallel with the small; in short, the stomach of a child resembles that of the carnivorous mammalia.

*Fig.* 2.

The form of the stomach of the adult is very different (*Fig.* 2): it is more circular; the œsophagus (*a*) is not inserted into the left extremity, as is the case with the child's, but into the middle between the left extremity and the pylorus (*b*). The pylorus itself is drawn back towards the cardia, and both brought very near to each other; on this account, the small curvature is very short (*ce,*) while the large curvature, on the contrary, is disproportionately extended (*dddd,*) forming not only the entire lower circumference of the stomach, but also surrounding that part of the fundus situated between the cardia and the left extremity; so that the large curvature alone forms about four-fifths of the whole circumference of the stomach. It must also be added, that the fundus does not pass into the pyloric portion gradually and gently, as is the case with the child's, but that the latter is separated from the former by a sort of neck or contraction (*cc*), sometimes more, sometimes less, strongly marked. In consequence of this the left part of the stomach assumes an almost circular form, and the whole very much resembles the form of the stomach of the rat or rabbit, although in a less marked degree than in these animals.

To each of these different forms of the stomach, an entirely distinct motion, peristaltic as well as antiperistaltic, has been given. In the child's stomach, where the small curvature is extended almost parallel with the large one, the food is expelled with nearly equal power by the undulating motion of both curvatures, and forced towards the pylorus by the peristaltic and towards the cardia and œsophagus by the antiperistaltic. In consequence of this, vomiting in children is very easy, because the œsophagus is situated at one extremity of the stomach, towards which the food is forced, at the same time that the pylorus closes and the cardia opens. But the process is very different in the stomach of the adult: in this, the small curvature is so much shortened, and the large one so much extended, that the food is not equally propelled from both sides, but the motion is almost confined to one side, and is effected principally by the large curvature, which embraces almost the entire circumference of the contents of the stomach; by this partial action, the contents of the stomach are moved rather in a rotary direction, which completely stops towards the contracted pyloric portion, turning round in the fundus from the left side to the right when urged by the peristaltic motion, and from the right to the left when by the antiperistaltic. In consequence of this, during the act of vomiting, the antiperistaltic motion does not direct the food towards the cardia and œsophagus, but merely communicates to it a motion contrary to that given by the peristaltic; and herein the reason is to be sought why, notwithstanding the pressure of the abdominal muscles and the diaphragm, the contents of the stomach are so difficult to be voided, and that, in many herbivorous animals, where the small curvature is still more shortened, the evacuation is impossible. The evacuation of the contents of the stomach of an adult can be effected only by a strenuous effort, produced by the strong pressure of the diaphragm and abdominal muscles at the same time that the œsophagus opens and shuts alternately; the stomach itself would be incapable from its antiperistaltic motion alone to discharge its contents upwards. In this respect there exists a completely different state of things in the pyloric and cardiac portions of the stomach. The pyloric portion from the point (*cc,*) where it is so much reduced in diameter, exhibits a more regular or intestine-like form of both curvatures, and the contents are on that account easily urged forwards into the duodenum; but, in the other direction, the contemporaneous motion of the two sides ceases beyond the contracted part, becoming, as already stated, rotary, in the cardial portion.

These details satisfactorily explain the differences so often referred to between children and adults. The former can discharge the contents of their stomachs by the antiperistaltic motion alone, without any perceptible assistance of the abdominal muscles; and the least pressure from these will increase the discharge. Animals whose stomachs are cylindrical, and in which, consequently, the ordinary relation between two curvatures entirely ceases, such as frogs or fishes, can, as it appears, with facility empty their stomachs by means of the antiperistaltic motion alone, without any cooperation of the abdominal muscles; and it is thus that they often throw up pieces of food merely on account of their inconvenient position in the stomach, and swallow them again in a more acceptable direction; even dogs after having swallowed a piece of bone frequently adopt a somewhat similar method. The human stomach in the earlier stages of its formation puts on the cylindrical form of the stomach of fishes and amphibious animals; in the embryo it appears only as a slight enlargement and elongation of the œsophagus in the abdominal cavity, with the cardia directed upwards and the pylorus downwards, as is the case with frogs. The stomach assumes its horizontal position only at a later period when the curvatures become developed.

There are naturally an endless number of transitions and intermediate stages of development, between the cylindrical, conical form of the stomach of the infant and that of the adult; and these numerous transitions will be accompanied by as many degrees of facility or difficulty in vomiting. What appears to me particularly interesting in a medical point of view is, that the round stomach of the adult is frequently seen in children of a diseased or merely of a disordered condition at a much earlier age than usual, and that such children also generally vomit with much more difficulty. I have had opportunities of making this observation in several post-mortem examinations of scrofulous children; and in one instance was able to describe before death the probable form of the stomach, from the extraordinary difficulty with which the child vomited. On the other hand, the fundus of the stomach of adults is not always found to extend, in a like degree, beyond the insertion of the œsophagus towards the left side. There are human stomachs with the fundus so much developed, as to be with difficulty distinguished from those of herbivorous animals; and others, again, which approach nearer to the form of the dog's stomach from their imperfect development.

The question naturally here suggests itself:—What is the cause, not only of these differences, but of the changes in general, to which the stomach is subject at different periods of life? To me it appears that the cause is principally to be sought in the nature and quantity of the food. The cylindrical form of the stomach in children continues only while they are fed on milk, consequently on purely animal food; as soon as they receive vegetable food in any quantity, the fundus begins to develop itself. On that account, even in the first year, a strong development of the fundus is found to have taken place in such children as have been weaned immediately after their birth and fed on soft pap made of flour, potatoes, or bread. The influence of the food on the form of the stomach is distinctly observable in older persons. The stomachs of such persons who live principally on potatoes and other vegetables are found to resemble most those of herbivorous animals; while the fundus in individuals who live more on rich animal food is less developed. I have shewn in my paper, '*De Alimentorum Concoctione*,' that the stomach of dogs and cats (animals purely carnivorous,) will assume the circular form after they have been fed for some time on messes of potatoes, meal, and bread; but that their stomach will retain its original oblong form if fed on animal food alone. On this account, the round form of the stomach observed in the domesticated carnivorous animals is never found in wild animals of the same class, such as, for example, the pole-cat.

Man, as an omnivorous animal, certainly possesses the type of the more rounded form of the stomach; but the extent of the development until it attain the form of the stomach of animals purely herbivorous, will, however, in a great measure, be determined by the degree of preponderance of vegetable over animal food; and the development may be increased till it become morbid. The reason why vegetable diet should develop the fundus to such a degree that the stomach assumes the circular form, (and the rotatory motion be in consequence given to its contents,) is, I believe, the following: I have shewn elsewhere, in speaking of animals, that vegetable food is of much more difficult digestion, and consequently is retained much longer in the stomach. The food requires to be moved about longer, and not immediately propelled into the intestine; hence the rotary motion, by which it is agitated in the stomach without being directly emptied into the pylorus. By this action the digested part of the vegetable food is gradually separated by layers on the surface of the mass, and is conducted into the pyloric division, in order to be passed into the intestine, while the undigested part continues in rotary motion in the centre of the stomach. In carnivorous animals the process is very different: the animal food, being soon digested, is directly propelled towards the pylorus by the united action of both curvatures, and does not require to undergo a prolonged rotary motion; whereas, if vegetable food be received in a stomach so constituted, it will necessarily pass into the intestine in a raw or only partially digested state. On the other hand, herbivorous animals cannot perfectly digest animal food unless the form of the stomach undergo a change, as, by long detention in the organ, the food, instead of being digested, becomes putrid. The attempts, therefore, which have been made in some places to feed sheep, horses, and oxen, on fish or other animal matter, must ever fail. The enquiry whether the stomach of these animals might not be transformed by gradually accustoming them to animal food, is foreign to the present subject. But, even with dogs and cats, experience shews that purely vegetable food does not succeed, as it almost invariably renders them subject to the mange, (*räude.*)—But, to return to the cause of vomiting in children and adults.

Although the form of the stomach plays the principal part in vomiting, there seems to be another agent strongly cooperating with it, namely, the sensibility of the organ itself, particularly in respect of the nausea or sickness which produces the motions of the stomach in the act of vomiting. This is the reason why I do not assert that lunatics, who generally vomit with so much difficulty, experience this difficulty only because they have a herbivorous stomach; in such a case, we must consider the state of the brain as well as the sensibility of the stomach; the torpidity of the brain being often such as not to admit the perception of nausea: these persons, perhaps, frequently do not vomit because they do not experience nausea.

We have been endeavouring to shew that the food is detained longer in the stomach of the herbivorous form, because it is kept longer in action there, without passing directly into the intestine, and that this form is adapted only to the more indigestible quality of vegetable food. If a stomach so constituted be suddenly filled with animal food, this food will be detained longer by the rotary motion than is necessary for the purpose of digestion, and the consequence will be, that the whole process will be disturbed, and the food, instead of being digested, will undergo a chemical decomposition. From this we may also conclude, that nothing will disorder the stomach sooner than sudden repletion with animal food after long use of a diet in which the vegetable preponderated. Excess of vegetable food is much less injurious in such cases, as undigested vegetable matter is, in the intestine, not so easily decomposed, and excites the peristaltic motion more than animal food. It follows that we ought carefully to avoid sudden change of diet from vegetable to animal. To this may be ascribed the greatest part of the gastric diseases prevalent in summer, and still more in autumn, when the stomach, after having been for some time accustomed to vegetable diet, is suddenly charged with large quantities of animal food.

The only remaining question is, whether we can produce excessive retching by larger doses of emetics, as a substitute for the want of peristaltic expulsory motion in persons having stomachs of the herbivorous form? On closer observation, however, we shall be induced to believe that large doses of emetics in such cases would fail in producing the intended effect. There are persons in whom very powerful emetics would sooner produce death than vomiting, as is the case with rabbits. In such cases, I think, the greatest assistance will be afforded by such means as will facilitate vomiting, by increasing the pressure of the abdominal muscles on the stomach, such as filling it with fluids, particularly gelatinous fluids, or any thing calculated to increase the elastic tension of the parts: perhaps, after all, the best means of facilitating vomiting in stomachs of such a conformation will be starch-flower or arrow-root boiled to a paste, as formerly recommended by Hufeland.—*Hufeland und Osann's Journal. März*, 1835.

---

### *On the Influence of the Nerves on the Development of the Muscular System.* By Professor ANTONIO ALESSANDRINI.

IN 1829, Professor Alessandrini published an account of the dissection of a calf whose medulla spinalis terminated about the tenth dorsal vertebra, and in which all the voluntary muscles were absent which are usually supplied by those spinal nerves which were wanting. Thus there were no muscles to the hinder limbs, and some of the muscles of the trunk were but half developed. On the other hand, all those parts essentially composed of cellular tissue, the integuments, adipose tissue, vascular system, aponeuroses, bones, &c. of the hinder extremities, were natural.

The conclusions which he then came to on the influence of the nerves in the growth of muscles were strengthened by the following case of monstrosity which has recently fallen under his notice. The vertebræ and spinal marrow of a young pig, removed from the uterus of a sow killed for food, who had gone with young her full time, were found to be deficient below the fifth dorsal vertebra. The head, neck, anterior part of the chest, and fore-legs were natural and muscular; but the posterior part of the thorax and abdomen had the appearance of a large ovoid bladder with strong aponeurotic walls, to whose fundus and lower extremity were attached the ossa innominata, which sustained the hinder limbs. All those parts of the thorax and abdomen, as well as of the hinder limbs, which were deprived of nerves, had no voluntary muscles. The viscera of the thorax and abdomen were natural, as well as the par vagum and grand sympathetic. The muscular coat of the intestines was very visible. Another instructive peculiarity was, the existence of an isolated portion of the vertebral column of the coxygeal or caudal region, containing a small cylindrical piece of the medulla spinalis, from which sprang some delicate nervous filaments distributed to muscular fibres, representing some portions of the caudal muscles.

From these two cases the Professor deduces the following propositions: 1. That the nerves contribute to the formation of muscular fibres more than the blood-vessels, as the latter were regularly formed in the hinder limbs. 2. Muscular fibre is not only formed by the influence of the nerves of animal, but also of organic life. 3. The existence of an isolated portion of the spinal marrow in the pig demonstrates that the various nodi or centres of the cerebro-spinal axis are formed independently of each other, so that it cannot be said that one is the production or prolongation of the other.

*Bulletino delle Science Mediche. Gennajo*, 1835. Vol. II. *Bologna.*

---

## Figures and Tables

**Fig. 1. f1:**
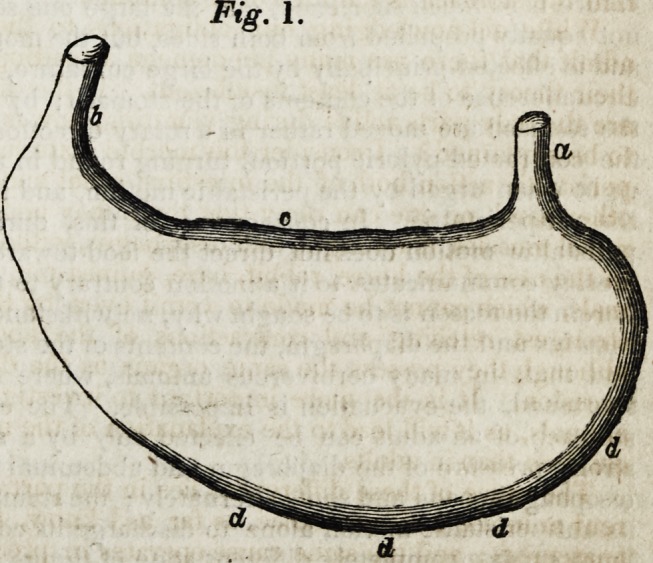


**Fig. 2. f2:**